# Mobilisation of jerboa kidney gene networks during dehydration and opportunistic rehydration

**DOI:** 10.1016/j.isci.2023.107574

**Published:** 2023-08-09

**Authors:** Benjamin T. Gillard, Nabil Amor, Fernando Alvira Iraizoz, Audrys G. Pauža, Colin Campbell, Michael P. Greenwood, Abdulaziz N. Alagaili, David Murphy

**Affiliations:** 1 Molecular Neuroendocrinology Research Group, Bristol Medical School: Translational Health Sciences, Dorothy Hodgkin Building, University of Bristol, Bristol, England; 2 LR18ES05, Laboratory of Biodiversity, Parasitology and Ecology of Aquatic Ecosystems, Department of Biology - Faculty of Sciences of Tunis, University of Tunis El Manar, Tunisia; 3 Department of Zoology, King Saud University, Riyadh, Kingdom of Saudi Arabia; 4 Department of Engineering Mathematics, Ada Lovelace Building, University of Bristol, Bristol, England

**Keywords:** Animals, Environment, Exposure, Transcriptomics

## Abstract

Desert animals have evolved systems that enable them to thrive under dry conditions. Focusing on the kidney, we have investigated the transcriptomic adaptations that enable a desert rodent, the Lesser Egyptian Jerboa (*Jaculus jaculus*), to withstand water deprivation and opportunistic rehydration. Analysis of the whole kidney transcriptome showed many differentially expressed genes in the Jerboa kidney, 6.4% of genes following dehydration and an even greater number (36.2%) following rehydration compared to control. Genes correlated with the rehydration condition included many ribosomal protein coding genes suggesting a concerted effort to accelerate protein synthesis when water is made available. We identify an increase in TGF-beta signaling antagonists in dehydration (e.g., GREM2). We also describe expression of multiple aquaporin and solute carrier transporters mapped to specific nephron segments. The desert adapted renal transcriptome presented here is a valuable resource to expand our understanding of osmoregulation beyond that derived from model organisms.

## Highlights

• The desert rodent *Jaculus jaculus* is an expert at surviving without water
• Large changes in renal gene expression respond to dehydration and rehydration
• BMP antagonist GREM2 may be important in the Jerboa kidney dehydration response
• Many genes that change after rehydration are involved in translation processes

## Graphical abstract

**Figure:**
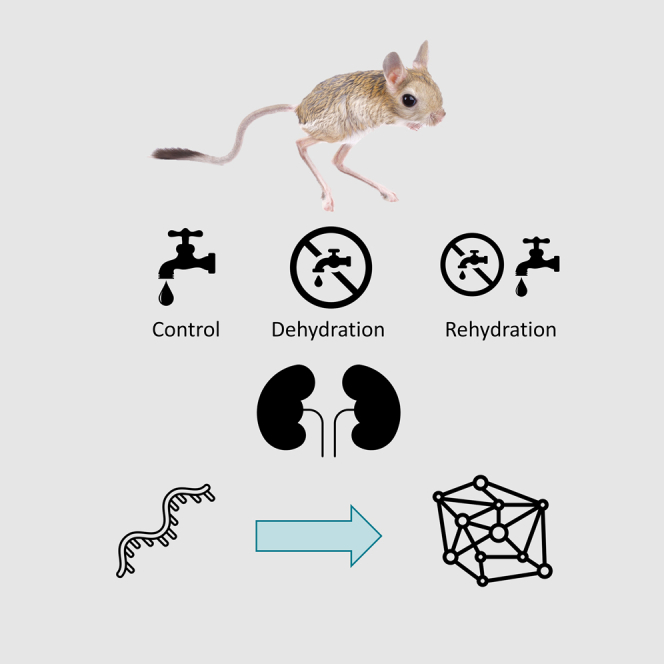

## Introduction

Climate change is affecting the water cycle^1^ including the availability of water. Desertification and extreme droughts are becoming more common and with this comes a significant physiological and health burden to both people and wildlife. Epidemics of kidney disease are being reported across the world linked to heat stress and poor hydration.^2^^,^^3^^,^^4^

Osmoregulation concerns the maintenance of internal fluid balance whereby water input relies on drinking, diet, and metabolic water and output is determined by urine, respiration, and sweat. In mammals, osmoregulation is tightly regulated using a system centered around the peptide hormone arginine vasopressin (AVP). During osmotic stress such as water restriction, a loss of extracellular water volume increases plasma osmolality as the solute (primarily sodium) concentration is increased. This stimulates the release of AVP from the posterior pituitary gland into the systemic bloodstream. In the kidney, AVP initiates a signaling cascade that reduces urine water loss by reabsorption of water from the collecting ducts via the water channel aquaporin 2 (AQP2)^5^ and others. A myriad of active and passive channels line nephrons, transporting solutes for the creation of an osmotic gradient. As the nephron descends into the renal medulla, the interstitial fluid becomes highly concentrated, allowing water to flow from the lumen to create urine with high osmolality, an ability belonging only to birds and mammals.^6^ When water intake increases during rehydration, the opposite action occurs to inhibit reabsorption of water, producing dilute urine. This process is continuous but is put under extreme pressure when water availability is reduced over a prolonged period.

Some animals have evolved to survive and thrive in hostile arid environments such as the deserts of the Kingdom of Saudi Arabia. Characterizing the response to dehydration (and rehydration) in a desert adapted mammal can identify essential mechanisms shared by all mammals as well as the unique specializations that have evolved in creatures that are able to survive long periods without water. The Lesser Egyptian Jerboa (*Jaculus jaculus*), hereafter referred to as the “Jerboa”, is a rodent that is found in abundance across the deserts of North Africa and the Arabian Peninsula. The Jerboa has an omnivorous diet of plants, seeds, and animals^7^ and this is presumably where it derives water from as, even in captivity, Jerboas remain healthy with no water access.^8^ This ability to survive without water makes the Jerboa a tractable and attractive model to investigate osmoregulatory mechanisms in desert adapted species. Previously, Khalil and Tawfic^9^ described kidney morphology and found increased relative medulla thickness in the Jerboa compared to the white rat. This has been replicated in other desert animals and is considered to have a positive correlation with urine concentrating ability (Munkácsi and Palkovits^10^; Beuchat^11^; al-Kahtani et al.^12^). A series of experiments in the Jerboa and its closely related cousin *Jaculus orientalis* in Morocco during the 1980s showed higher basal plasma vasopressin, higher urine osmolality, and a greater ability in the kidney to retain water than Wistar rat counterparts (Baddouri et al.^13^^,^^14^). With a genome recently assembled as part of the Genome 10K initiative,^15^ the molecular basis of these highly adapted mechanisms can now be studied.

We first asked how physiological markers of osmoregulation respond to extended water restriction (beyond the survival limits of many mesic mammals) and rehydration in the Jerboa. Then, we cataloged the renal transcriptomes of Jerboas under euhydrated, dehydrated, and rehydrated conditions. Comparison of these datasets enabled us to identify transcriptomic changes caused by these transitions. Network analysis grouped correlated genes together into modules which were then linked to physiological traits thus revealing functionally relevant gene clusters and “hub” genes that influence the Jerboa’s ability to thrive in the desert.

## Results and discussion

### Experimental design

**Figure 1:**
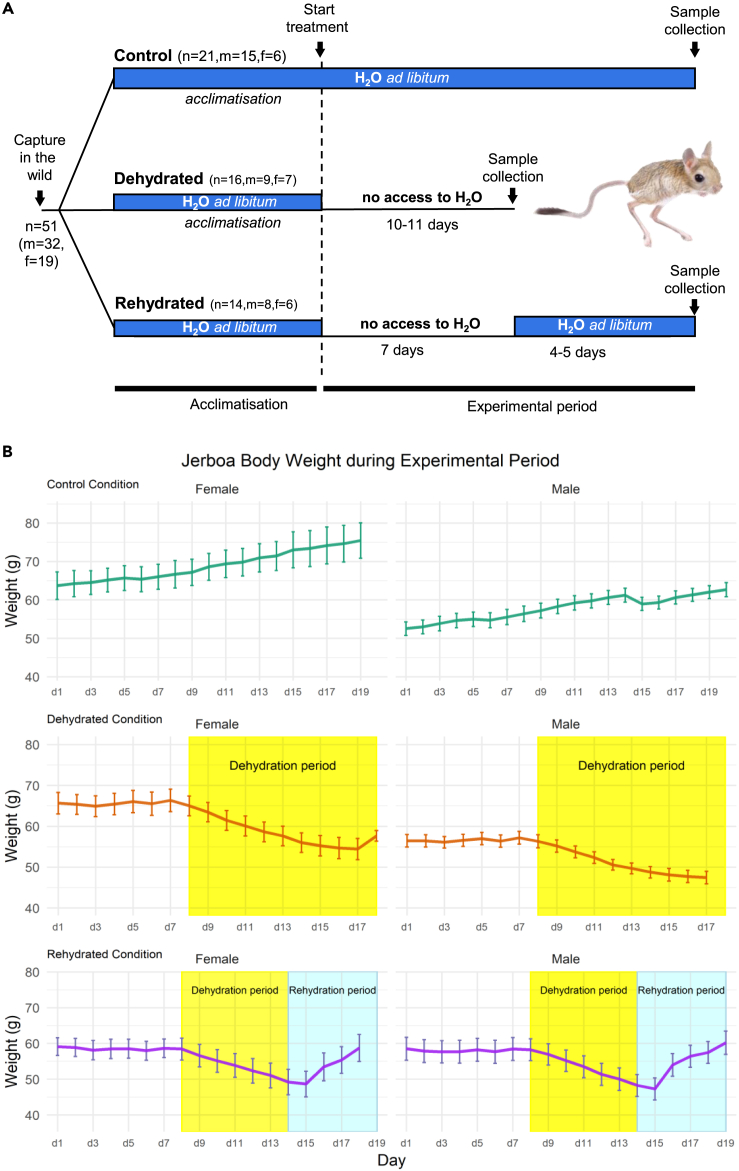
Experimental design and blood analytes in the Jerboa (A) Wild caught Jerboa were split into three experimental groups: Control (free access to water), dehydration (water restriction for 10/11 days), or rehydration (water restriction followed by free water access). An acclimatization period was used to control for any differences between animals diet and water access in the wild. (B) Total body weight (in grams) of Jerboas throughout the experimental period and error bars representing SEM. Male and female animals are separated onto different plots due to the significant influence of sex on body weight. Days during the water restriction period are highlighted in yellow. Days during the rehydration period are highlighted in blue.

Wild caught Jerboas were split into three experimental groups: control (free access to water), dehydration (water restriction), and rehydration (water restriction followed by free water access). At the end of the experimental period, serological samples were taken for blood analyte measures. Whole kidneys were collected for transcriptome determination by RNA sequencing (RNAseq) analysis. Network analysis of transcriptomic data were used to identify gene networks and hub genes. Summarized in Figure 1A.

### Physiological markers of hydration

A general linear model (GLM) was used to estimate the effect of the experimental conditions on physiological markers while incorporating possible confounding variables (sex, lipemia, hemolysis). Each model was reduced based on Akaike information criterion with the final model reported as formula, adjusted R^2^, test statistic (degrees of freedom), and significance value. Weighted mean differences (±SE) are reported for dehydration and rehydration conditions compared to control (and female animals where relevant). A full description of models can be found in Table S1.

#### Weight

Weight loss is a classical consequential marker of water loss due to dehydration.^16^ To investigate how weight changes in Jerboas responding to dehydration and rehydration, weight was recorded daily throughout the experimental period (Figure 1B). To ensure there was no bias within the condition groups, a GLM with condition (experimental group) and sex as independent variables was performed using the caught weight as the dependent variable. It showed that the only significant influence on the body weight of Jerboas when caught (∼Condition+Sex, 0.17, F[47] = 4.36,p = 0.01) was sex with male Jerboas weighing less than female Jerboas (−8.89 ± 2.82, T[38] = -3.154, p = 0.003). A reduced GLM (∼Condition+Sex, 0.46, F[47] = 15.29,p=< 0.001) with dissection weight as the dependent variable showed both dehydration (−17.19 ± 2.58, T[47] = -6.65, P=< 0.001) and rehydration (−8.04 ± 2.661, T[47] = -3.022, p = 0.004) groups had significantly reduced weight compared to controls (Table 1).

**Table 1:** Summary of analyte means (with SEM)

	n	Range	Control	Dehydration	Rehydration
Caught_Weight	51	29,70	54.1 (1.89)	57.4 (2.14)	54.66 (2.774)
Weight_7DayDehydration	45	38,82	63.0 (2.51)	52.4 (1.64)∗∗∗	48.65 (2.225)∗∗∗
dissection.weight	51	40,84	66.8 (1.83)	50.8 (1.68)∗∗∗	59.58 (2.390)∗∗
Sodium	44	128,159	142.9 (0.75)	146.7 (1.29)∗∗	137.36 (1.216)∗
RBC	41	6.4,16	12.4 (0.40)	14.2 (0.19)∗∗	12.43 (0.270)
HGB	41	7.7,25	17.3 (0.59)	20.6 (1.17).	18.20 (0.380)
HCT.	41	11,54	41.8 (1.45)	47.2 (1.58)	38.73 (2.281)∗
Calculated_Osmolality	44	273,374	304.2 (1.38)	315.2 (4.98)∗	295.07 (3.240)

Signif. codes: 0 ‘∗∗∗***’ 0.001 ’∗∗****’ 0.01 ’∗*’ 0.05 ‘.’ 0.1

Summary of selected analytes of interest including number of animals included (n), range of measurements, and the mean value from each condition group (with SEM in brackets). Significance markers are from general linear models for each analyte (full model information can be found in Table S1) as this analysis can control for external variables such as sex. Caught weight, 7 days dehydration weight, and dissection weight measured in grams. Sodium mmol/l. RBC Red Blood Cell Count (10ˆ12/L). HGB Hemoglobin (g/dL). HCT. Hematocrit (%). Calculated Osmolality (mOsm/kg).

#### Blood analytes and hematology

Table 1 shows the means (and SEM) of selected analytes in each condition group. The results of GLM analysis for each analyte are presented here and significance results are included within Table 1. Full details of all GLMs can be found in Table S1. Sodium (∼Condition+Lipemia+Hemolysis,0.57,F(33) = 13.38,p < 0.001) increased after dehydration (4.15 ± 1.41, T(33) = 2.95, p = 0.006) and decreased after rehydration (−3.54 ± 1.62, T[33] = −2.184, p = 0.036). However, hemolysis (p = 0.034) and to lesser extent lipemia (p = 0.066) both had a negative influence on sodium. Calculated osmolality (∼Condition+Hemolysis,0.29,F[34] = 6.14,p = 0.002) incorporates sodium, glucose, and urea nitrogen and was increased after dehydration (11.66 ± 5.00, T[34] = 2.331, p = 0.026) but not rehydration (−7.47 ± 5.34, T[34] = −1.40, p = 0.171) compared to control. Modeling showed red blood cell count (∼Condition+Sex+Lipemia,0.26,F[29] = 3.947,p = 0.011) increased after dehydration (1.71 ± 0.5,T[29] = 3.414,p = 0.002) then recovered after rehydration (−0.16 ± 0.37,T[29] = -0.421,p = 0.677) compared to control. Alongside this, Hematocrit % (∼Condition+Sex+Lipemia,0.2,F[29] = 2.992,p = 0.035) trended upward non-significantly following dehydration (4.68 ± 3.21,T[29] = 1.457,p = 0.156) and was significantly reduced after rehydration (−5.14 ± 2.37,T[29] = -2.166,p = 0.039) compared to control. Both red blood cell count and hematocrit % are inversely proportional to the water content in the blood.

Markers of note that were not influenced by condition included creatinine, urea nitrogen, glucose, potassium, and total protein. (Table S1).

Blood sodium concentration is a commonly used biomarker for hydration. In Jerboas, we saw an increase in sodium concentration following dehydration and a decrease significantly below control following rehydration, as has also been observed in other desert rodents^17^ and is a typical response often reflecting a loss of extracellular water causing an increase in solute concentration. Further to this, a significant increase in red blood cell count and calculated osmolality during dehydration suggests a reduction in plasma water content. These changes then recover after rehydration, and in the cases of hematocrit % and sodium, levels are significantly less than control. These responses are canonical in a variety of mammals, with dehydration induced increase in sodium and plasma osmolality recorded in chickens,^18^ camels,^19^ horses,^20^ and dogs.^21^ A study with a similar dehydration time in another desert rodent, the Kangaroo Rat (*Dipodomys*), also described a significant decrease in body weight and an increase in plasma sodium.^22^

Not all analytes are as predictable in response to dehydration. Creatinine is often used as a surrogate marker for kidney function as a rise in creatinine is associated with a fall in glomerular filtration rate, which has been observed in dehydration in mammals.^19^^,^^20^^,^^23^ In this study, creatinine did not significantly change with dehydration (or rehydration). This mirrors the results in the cactus mouse^17^ where the same measurement technology was used for a comparison of dehydrated and euhydrated animals. The difference in responses between xeric and mesic animals may suggest special protective mechanisms for kidney function in the former animals that routinely face osmotic stress.

The physiological markers presented here confirm the experimental design is appropriate to illicit the osmoregulatory response in Jerboas. The results also confirm that canonical responses are present across diverse habitats.

#### Transcriptome dynamics

Using RNAseq, we investigated how the Jerboa’s kidney transcriptome behaves when water is restricted and how it recovers when water becomes available again. The complete transcriptome datasets are presented in Table S2, have been banked for access (GSE225470), and can be visualized using the publicly available web application (https://app-shiny.services.bris.ac.uk/JerboaKidneyGeneSearch/).

**Figure 2:**
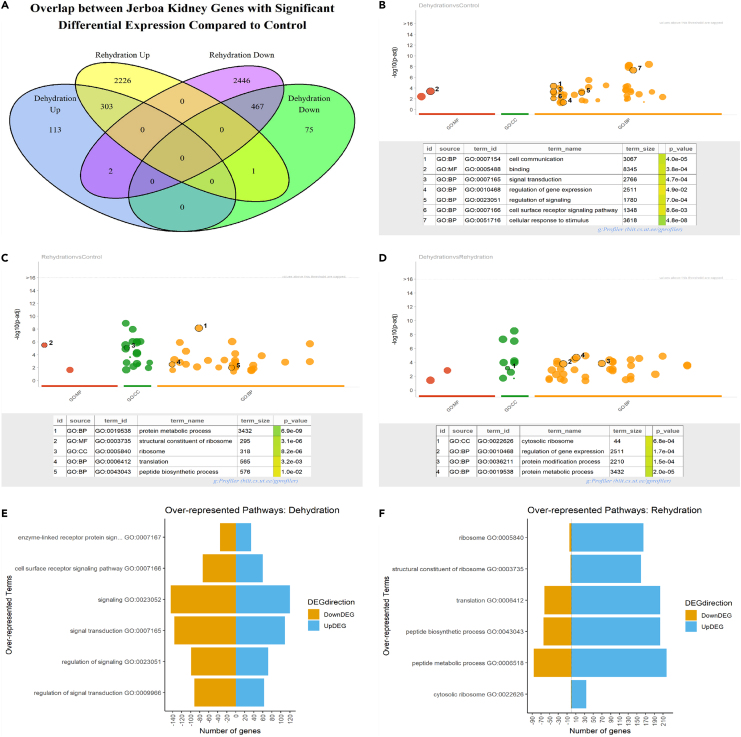
Transcriptomic analysis of the Jerboa kidney (A) Venn diagram showing the number of DEGs (padj < 0.01) and their direction of regulation compared to control. (B–D) GSEA analysis for DEGs regulation after dehydration compared to control, rehydration compared to control, and rehydration compared to dehydration respectively. Selected terms of interest are highlighted in the table below the Manhattan plot. (E and F) Plots of selected over-represented GO terms highlighted by GSEA after dehydration (E) and rehydration (F) in comparison to control. The count of genes downregulated is shown in orange on the left of the plot. The count of upregulated genes is shown in blue on the right of the plot.

A total of 961 (6.4% of detected genes) differentially expressed genes (DEGs, padj < 0.01) were regulated in response to dehydration and 5445 DEGs (36.2% of detected genes) were regulated after rehydration in the Jerboa kidney compared to controls (Figure 2A). When comparing the dehydration group to the rehydration group, 2767 DEGs (18.4% of detected genes) were found. It is noteworthy that the number of DEGs in rehydration compared to control was 5.7 times greater than the number of DEGs in dehydration compared to control. This suggests the Jerboa kidney’s transcriptomic adaptations are more active when the perhaps uncommon opportunity to drink arises.

Many genes were regulated in specific conditions only. 113 DEGs were upregulated in dehydration only compared to control. 75 DEGs were downregulated only in dehydration compared to control. DEGs specific to rehydration were even more abundant. 2226 DEGs were upregulated in rehydration only compared to control. 2446 DEGs were downregulated in rehydration only compared to control. There was some directional overlap between DEGs regulated in dehydration and rehydration. 303 DEGs were upregulated in dehydration and rehydration compared to control. Of these, 51 were significantly upregulated in rehydration compared to dehydration. 467 DEGs were downregulated in dehydration and rehydration compared to control. Of these, 210 were significantly downregulated in rehydration compared to dehydration (Figure 2A).

### Gene Ontology

To investigate possible functional implications of identified DEGs in the Jerboa kidney, gprofiler2^24^ was used for Over Representation Analysis (ORA). There were 43, 46, and 50 over represented terms (< 0.05 padj) in the GO:MF, GO:CC, and GO:BP categories relating to the DEGs that were regulated in response to dehydration vs. control, rehydration vs. control, and rehydration vs. dehydration, respectively.

Terms enriched in DEGs in the dehydration vs. control comparison cell communication (GO:0007154, padj = 4.0e-05), regulation of signaling (GO:0023051, padj = 7.0e-04), and regulation of gene expression (GO:0010468) (Figure 2B). Enriched terms from DEGs in the rehydration vs. control comparison included translation (GO:0006412, padj = 3.2e-03) and peptide biosynthetic process (GO:0043043, padj = 1.0e-02) (Figure 2C). The rehydration vs. dehydration comparison DEGs were enriched for GO terms overlapping those found in the other comparisons such as regulation of gene expression (GO:0010468, padj = 1.7e-04) and protein metabolic process (GO:0019538, padj = 1.98e-05) (Figure 2D).

The directionality of genes within some of the enriched terms was explored further. Figure 2E shows the number of gene transcripts encoding proteins involved in signaling that were either up or down regulated in dehydration compared to control, showing a slight tendency for genes to be downregulated. There was a more pronounced trend in genes included in GO terms linked to translation to be upregulated after rehydration compared to control (Figure 2F). This suggests that changes in cellular output might be regulated at the level of translation following dehydration.

### Network analysis

**Figure 3:**
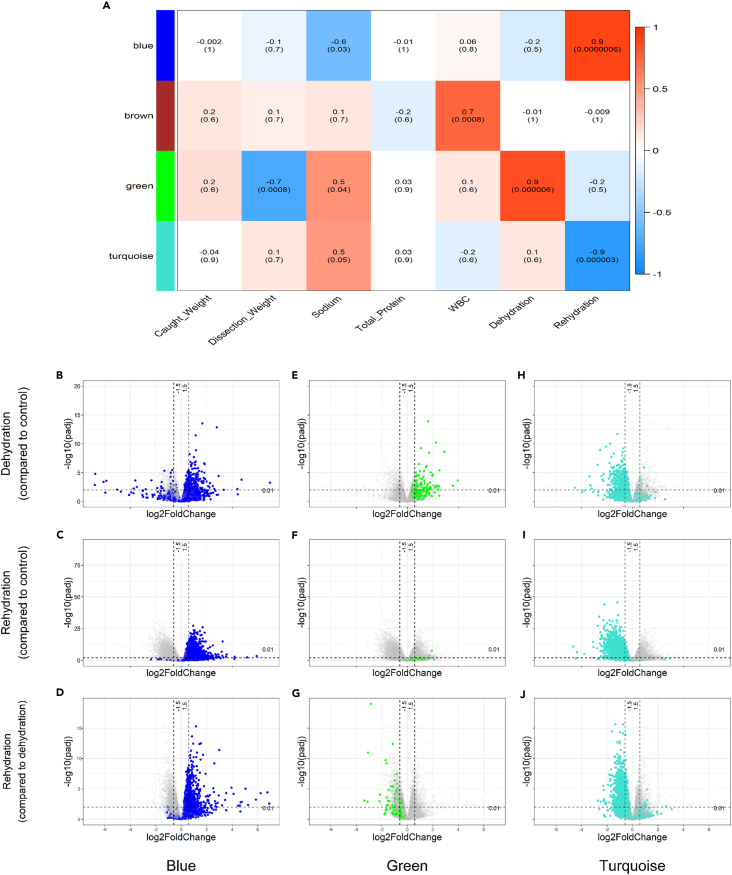
WGCNA modules and their relationship with external traits (A–J) Modules identified by WGCNA (y axis) and their correlations with data from selected traits. Correlations estimated by bicor with significance values in brackets below. Positive correlation is represented by red shading while negative correlation is represented by blue shading. Caught Weight and Total Protein were not influenced by the experimental conditions and so act as negative controls, showing that the design of the experiment is driving the expression of genes within modules. Volcano plots show the expression patterns for the “blue” (B–D), “blue” (E–G), “green” (H–J), and “turquoise” (L and M) modules after dehydration (B, E, H, and K) and rehydration (C, F, I, and L) compared to control and rehydration compared to dehydration (D, G, J, and M). Each data point represents a gene. Genes not in the module are gray. Dotted lines represent padj 0.01 horizontally and 1.5-fold change vertically. WBC, white blood cell count.

We identified a daunting number of osmotically regulated DEGs in the Jerboa kidney. Our initial analysis involved only genes that were below an arbitrary significance threshold (0.01 padj) and were restricted to pairwise comparisons between condition groups. To highlight specific genes, pathways, and networks that may be physiologically influential, we sought to categorize the data in an unbiased, data driven manner relative to physiological dynamics. We explored network tools that are based on the correlation of gene expression throughout the three experimental conditions and used a soft threshold to include as many relevant genes as possible. WGCNA is a tool that clusters genes together into modules (arbitrarily assigned colors) based on the correlation of their expression across all samples. These modules (represented by an eigengene) can then be linked to traits of interest (Figure 3A) with strong positive correlation to traits shown in red and strong negative correlations shown in blue. Four modules were constructed by WGCNA (Figure S1). This is fewer than other studies using WGCNA and is likely because of the experimental conditions being a very strong driver of expression as seen by the number of genes differentially expressed.

#### “Blue” module

The “blue” module consisted of 4730 genes. The “blue” module eigengene was positively correlated with the rehydration condition (bicor 0.9, p=< 0.001) and negatively correlated with sodium (bicor −0.6, p = 0.03) (Figure 3A). Enriched GO terms for the “blue” module included ribosome (GO:0005840, padj = 1.787e-28), translation (GO:0006412, padj = 1.227e-06), and structural constituent of ribosome (GO:0003735, padj = 4.983e-29). The similarity with terms found in the DEG list after rehydration reflects the upregulation of many genes in the “blue” module after rehydration. In the “blue” module, 6.9% (n = 328) of genes were DEGs after dehydration and 43.8% (n = 2071) were DEGs after rehydration compared to controls with all genes upregulated after rehydration (Figures 3B–3D).

Many of the genes linked to the translation GO term encode ribosomal proteins, which are conserved across species.^25^ Of the 82 RPL/RPS (ribosomal protein large/small families) genes included in the WGCNA, 85.4% of them were in the “blue” module. Mitochondrial RPs continued this trend with 90.7% of the 43 detectable genes part of the “blue” module. Translation was also a focus of GO terms that were upregulated during rehydration. The altered expression of these ribosomal genes is currently an area of intense interest; heterogeneous ribosomes can preferentially translate specific mRNAs.^26^ Thus, these genes may drive recovery from the stresses of water restriction by directing the expression of required proteins.

#### “Green” module

Sodium had a positive correlation with the “green” (bicor 0.5, p = 0.04) module eigengene (Figure 3A) and a strong negative correlation with dissection weight (bicor −0.7, p = 0.0008). The “green” module was enriched for a single term: extracellular region (GO:0005576, padj = 2.664e-03). The “green” module contained 25.4% (n = 87) DEGs, all of which were upregulated after dehydration, and 3.2% DEGs after rehydration which were all upregulated. 14.9% (n = 51) of “green” module genes were downregulated DEGs when comparing rehydration to dehydration (Figures 3E–3G). The “green” module contains genes that are expressed during water restriction, perhaps important to membrane transport.

#### “Turquoise” module

The “turquoise” module was made up of 4969 genes. The “turquoise” module eigengene was negatively correlated with the rehydration condition (bicor −0.9, p=< 0.001) and positively correlated with sodium (bicor 0.5, p = 0.05) (Figure 3A). The “turquoise” module was associated with the plasma membrane region (GO:0098590, padj = 3.896e-06) with enriched terms including transporter activity (GO:0005215, padj = 2.094e-03) and transmembrane transport (GO:0055085, padj = 9.002e-04) alongside chemical homeostasis (GO:0048878, padj = 4.814e-03). Many genes in the enriched terms belong to the solute carrier transporter (SLC) superfamily, which transport a wide variety of substrates: these are discussed in further detail in the following text. DEGs made up 10% (n = 496) of the “turquoise” module after dehydration and 52.6% (n = 2612) after rehydration, almost all of which were downregulated compared to control. 28.8% (1432) of “turquoise” module genes were DEGs when comparing rehydration to dehydration (Figures 3H–3J).

The two largest identified modules, “blue” and “turquoise”, contained many genes that were regulated in the same direction in both dehydration and rehydration compared to control suggesting a lasting response to water restriction in the Jerboa kidney. It is interesting to note that genes in these modules outnumber those in the module that showed recovery of expression to control levels after rehydration (i.e., “green”). It is possible this highlights differences between acute and chronic responses whereby genes in the “green” module, including well studied osmotically responsive genes such as AQP2 and AQP3, respond to the immediate need for water retention in the kidney. However, a longer-term transcriptomic response is more prevalent in the Jerboa, owing to their habitual lack of water.

#### “Brown” module

The “brown” module eigengene did not correlate with any of the blood analytes but did positively correlate with white blood cell count (WBC, bicor 0.7, p = 0.0008). However, there were missing data for these markers that may affect the strength of these results. Despite containing only a single DEG, the “brown” module was enriched for many immune related terms (Figure S1). A heatmap showing the expression of genes within the “brown” module highlighted samples CM01 and RHM01 as drivers of variance in this module, and a higher expression of inflammatory related genes in these two samples (Figure S1), suggesting that these individual wild-caught animals may have been carrying some unidentified pathology. The samples highlighted were those that clustered away from their experimental groups in the sequencing data PCA plot. When there are potential confounding variables that cannot be controlled for, such is the case when using wild animals, we have demonstrated how WGCNA can be used to describe variable samples and the genes that drive their variability.

#### “Hub” genes

**Figure 4:**
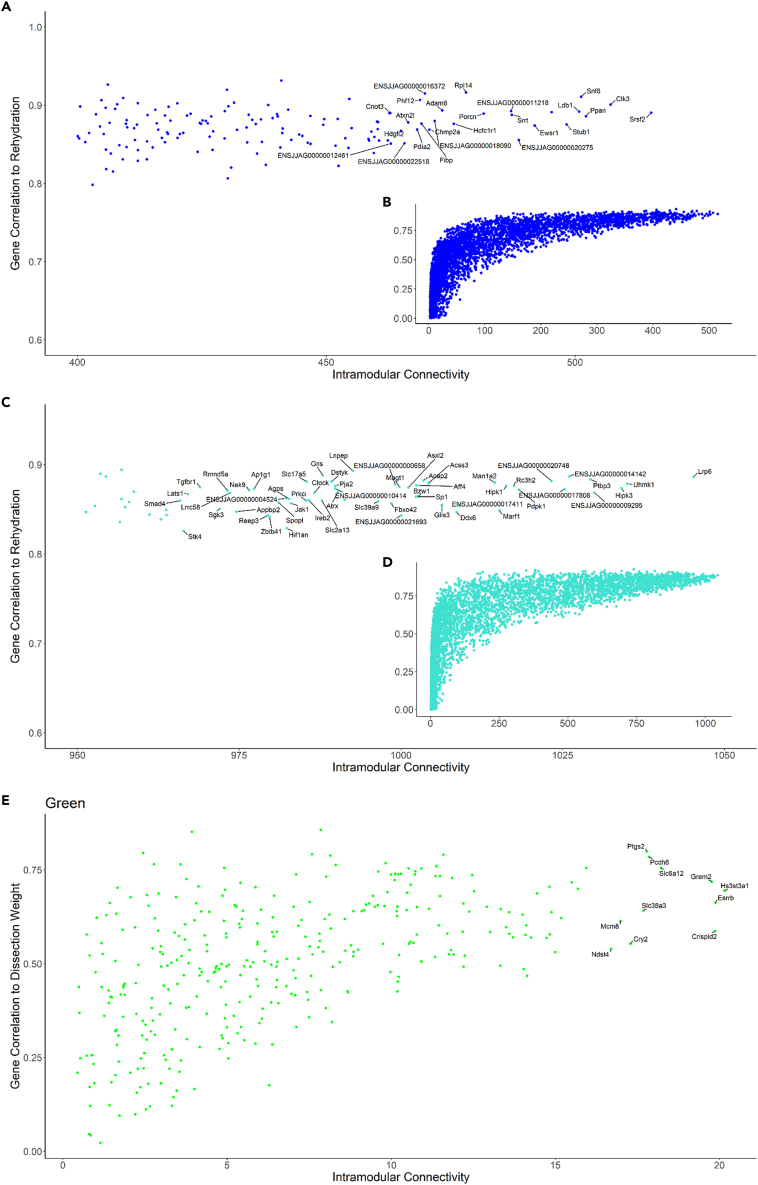
Intramodular connectivity plots for each module of interest (A) Genes in the “blue” module with high connectivity had a strong correlation to the rehydration condition. Genes with the highest connectivity are labeled. (B) The inset plot (B) shows the distribution of genes where most genes have low connectivity to other genes within the module. (C) Genes in the “turquoise” module with high connectivity had a strong correlation to the rehydration condition. Genes with the highest connectivity are labeled. (D) The inset plot (D) shows the distribution of genes within the turquoise module. (E) Genes in the “green” module with high connectivity had a strong correlation to dissection weight. Genes with the highest connectivity are labeled.

The identified modules were next investigated as discrete networks to find “hub” genes with high connectivity. Connectivity is a measure of neighbor genes closely correlated by expression patterns. The distribution of connectivity for genes within each module suggests the presence of a small proportion of highly connected “hub” genes (see distributions in Figure 4). Assuming genes within a module are functionally related, these “hubs” may be important central nodes in the response of each module and therefore targets for functional evaluation in the kidney. Selected “hub” genes for each module that have potential for future investigation are described.

A positive correlation was seen between gene significance (to traits of interest specific to each module) and intramodular connectivity (Figure 4). In the “turquoise” module, a gene with one of the highest within-module connectivity values was UHMK1 (KIS/ENSJJAG00000015993). UHMK1 is a kinase found to phosphorylate splicing factors (Manceau et al.^27^) and to interact with the translation initiator eEF1A.^28^ Interestingly, UHMK1 negatively affected the expression of AQP1 in mouse pituitary cells^29^ and the expression of UHMK1 and AQP1 are negatively correlated after dehydration in the Jerboa kidney. The list of “turquoise” genes ranked by connectivity was enriched for terms including protein kinase activity (GO:0004673, padj = 4.368e-03), protein modification process (GO:0036211, padj = 1.430e-03), and Golgi apparatus (GO:0005794, padj = 5.936e-04). There may be other targets included in the “turquoise” module worthy of investigation with regards to the protein pathways in recovery to osmotic challenge. High connectivity genes in the “blue” module involved in splicing and translational control include EWSR1 (ENSJJAG00000003308), CLK3 (ENSJJAG00000008106), SRSF2 (ENSJJAG00000013794), and LUC7L3 (ENSJJAG00000011218). A ranked list (by connectivity) of “blue” module genes was enriched for many GO terms relating to translation (GO:0006412, padj = 3.445e-20) such as cytosolic ribosome (GO:0022626, padj = 3.761e-17) and peptide biosynthetic process (GO:0043043, padj = 3.363e-19). This continues the theme of the “blue” module being important in translational processes. In the “green” module, genes with the high within-module connectivity included receptors (ESRRB/ENSJJAG00000007076) and channels (SLC6A12/BGT1/ENSJJAG00000018463, SLC38A3/ENSJJAG00000001766). A ranked (by connectivity) list of genes in the “green” module had no enrichment of GO terms.

### Canonical pathways in the kidney

**Figure 5:**
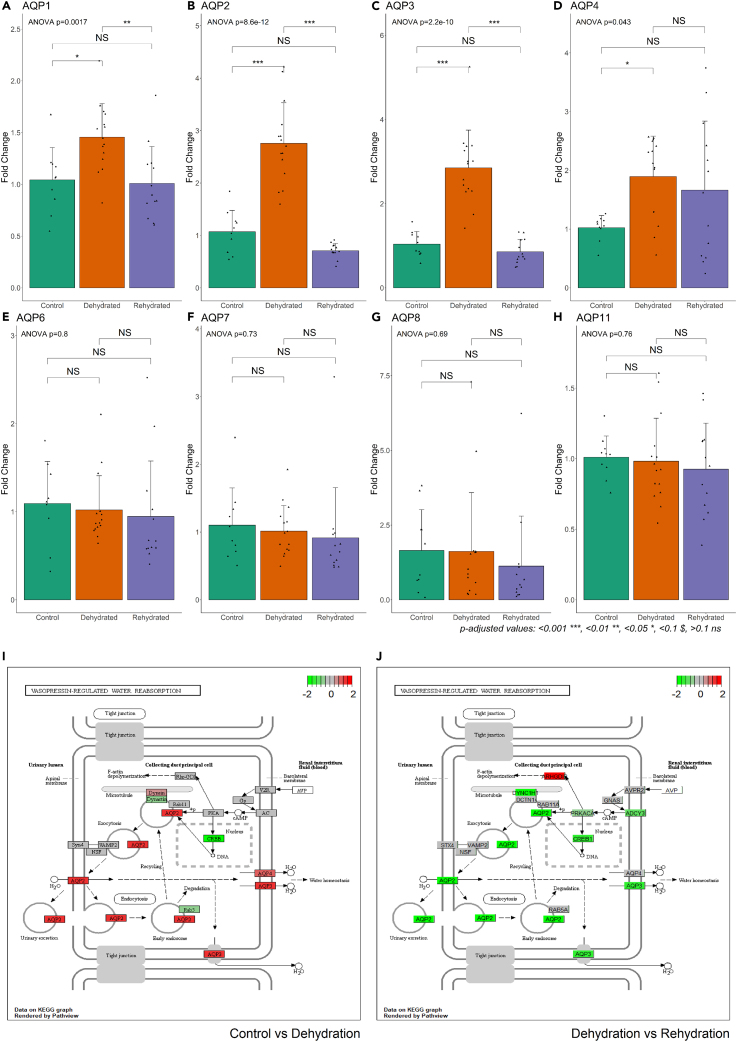
Expression of the Aquaporin gene family (A–I) RT-qPCR was used to generate data for all Jerboa samples (n = 42). Error bars represent standard deviation. Significance calculated by ANOVA followed by Tukey Honest Significant Differences for between group differences. Points represent individual samples. Male samples are shown as triangles, females samples are shown as squares. (I and J) KEGG pathway 04962 Vasopressin Regulated Water Reabsorption in dehydration compared to control (I) and rehydration compared to dehydration (J). Data for pathways are log2 fold change calculated by DESeq2 using RNAseq expression data. Human KEGG pathway was used whereby orthologue genes in human were found from Jerboa ENS IDs by the gConvert function as part of the gProfiler2 R package.

To compare the new Jerboa kidney transcriptome to canonical pathways involved in dehydration and rehydration, groups of genes with substantial previous research in model organisms are highlighted. Targets were identified in the RNAseq data then validated using qPCR and a larger sample number including female animals (Figure 5). Data shown are the relative expression between conditions generated from qPCR assays.

### Aquaporins

Aquaporins, a family of water channels, are an integral part of the dehydration response in the mammalian kidney. The process of reabsorbing water in the collecting duct and the involvement of AQPs is shown in Figures 5J and 5K. In the Jerboa kidney, AQP1, 2, and 3 were seen to be regulated between conditions as confirmed by qPCR (Figures 5A, 5B, and 5D). AQP2 and AQP3 shared a large increase in expression during dehydration, which was recovered back to control levels in rehydration. This is akin to the response in other mammals from both xeric and mesic habitats.^30^^,^^31^ The regulation of AQP1 also followed this pattern but to a lesser extent (Figure 5A). However, there was a discrepancy between AQP1 RNAseq and qPCR where RNAseq did not reveal a significant change between control and dehydration. Interestingly, AQP1 was non-significant across the three conditions in female animals (ANOVA female p = 0.11, male p = 0.0012). This may explain why the AQP1 qPCR data (including both male and female samples) did not confirm the RNAseq data and perhaps shows potential sex differences in the kidney response to dehydration in Jerboas. In rats, sex differences have been shown in renal AQP1 expression^32^ and some dehydration induced plasma markers.^33^ AQP4 followed a similar trend with both methods but did not reach significance in the qPCR assay (Figure 5D). AQP6, 7, 8, and 11 did not change between conditions.

Expression of AQP1, 2, and 3 showed positive correlation (bicor 0.79, 0.75, 0.58, respectively) with plasma sodium at an adjusted significance of (p = ) 0.01, 0.02, and 0.09, respectively. Both AQP2 and AQP3 were part of the “green” module (which was positively correlated with sodium and calculated osmolality) and were strongly correlated with each other. Under the assumption that strongly correlated genes are functionally similar and are part of the “green” module that appears driven by osmotic pressures, other genes with strong expression correlations with these aquaporins may be similarly influential in the kidney.

**Figure 6:**
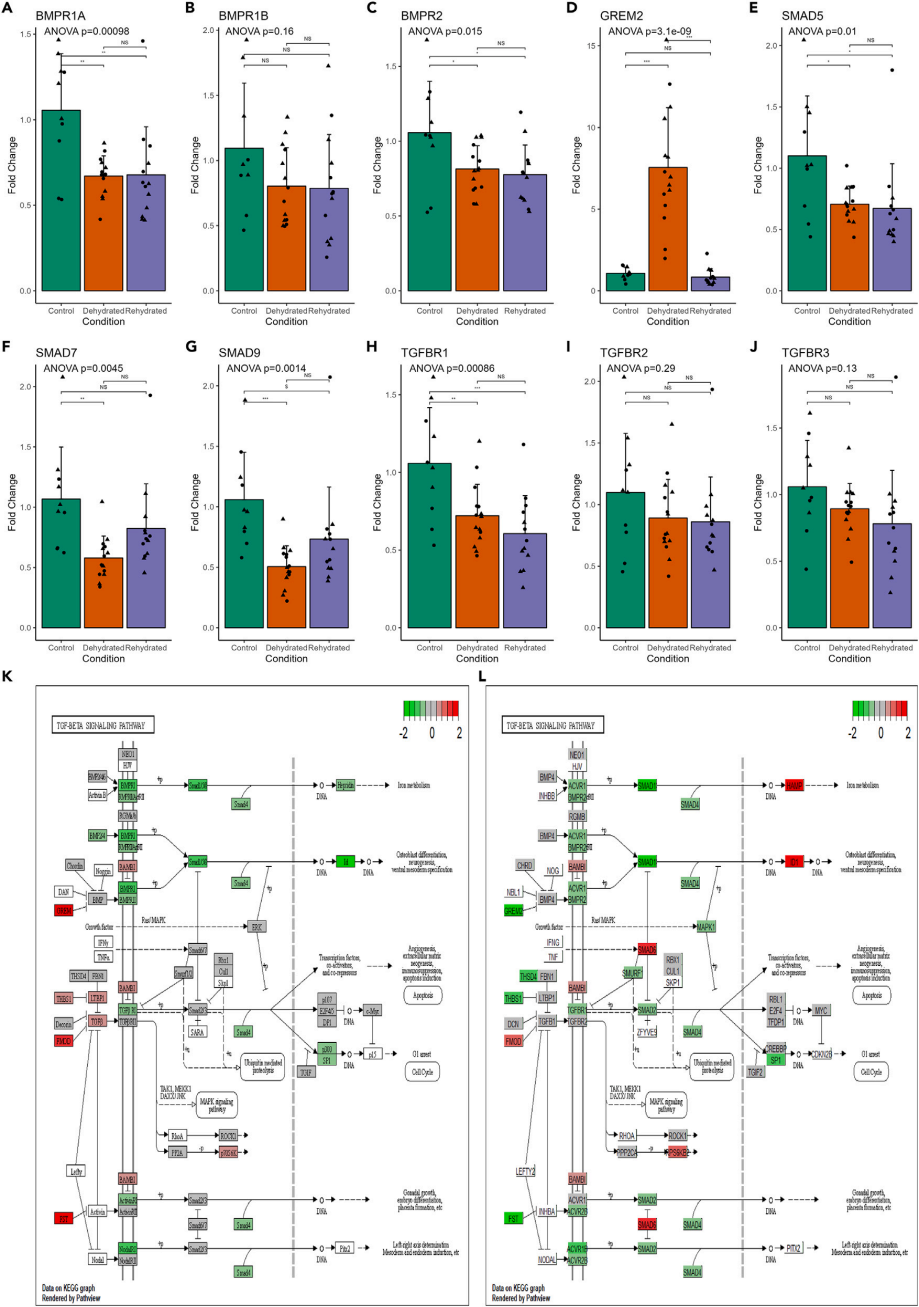
Expression of selected genes downstream of the BMP antagonist GREM2 (A–J) RT-qPCR was used to generate data for all Jerboa samples (n = 42). Error bars represent standard deviation. Significance calculated by ANOVA followed by Tukey Honest Significant Differences for between group differences. (K and L) KEGG pathway 04350 TGF-beta signaling pathway in dehydration compared to control (K) and rehydration compared to dehydration (L). Data for pathways are log2 fold change calculated by DESeq2 using RNAseq expression data. Human KEGG pathway was used whereby orthologue genes in human were found from Jerboa ENS IDs by the gConvert function as part of the gProfiler2 R package.

The closest neighbor gene to both AQP2 and AQP3 in the “green” module network by weighted correlation was GREM2 (ENSJJAG00000010680), which was also one of the highlighted “hub” genes (Figure 4E). GREM2 has been shown to be a BMP (Bone morphogenic protein) antagonist.^34^ In the Jerboa kidney, an upregulation of GREM2 during dehydration appears to coincide with a downregulation of BMP and TGF-beta receptors (Figure 6, BMPR1A/ENSJJAG00000021938, BMPR2/ENSJJAG00000015151, TGFBR1/ENSJJAG00000008037). These receptors continued to be downregulated in rehydration compared to control (Figure 6). BMPR1B (ENSJJAG00000012811), TFGBR2 (ENSJJAG00000009257), and TGFBR3 (ENSJJAG00000017313) were not differentially regulated. These receptors are upstream of the highly conserved SMAD family of transcription factors.^35^ SMAD5 (ENSJJAG00000019065), 7 (ENSJJAG00000017342), and 9 (ENSJJAG00000010404) were all downregulated in dehydration with SMAD7 and 9 recovering after rehydration (Figure 6). All the BMPR and TGFBR genes were part of the turquoise module along with 5 SMAD genes (SMAD1, 3, 4, 5, 9) suggesting a longer-term expression pattern than GREM2. This pathway is also involved in other regulatory mechanisms. For instance, GREM2 overexpression increases phosphorylation of SMAD 2/3 and decreases phosphorylation of SMAD 1/5/8 in podocytes (Wen et al., 2019). Many BMP antagonists have been linked to kidney disease states.^36^ In fact, GREM2 overexpression increases apoptosis in cultured human podocytes.^37^

In a desert dwelling animal such as the Jerboa, this must be important to avoid so it is surprising to see such a stark upregulation of GREM2 during dehydration. In the cactus mouse, GREM2 was also upregulated during water restriction although not significantly after multiple testing correction.^38^

We further investigated the expression of other BMP antagonists in the Jerboa kidney RNAseq data. USAG1 (SOSTDC1/ENSJJAG00000010091) and Follistatin (FST/ENSJJAG00000014889) followed a similar expression pattern to GREM2. Related members of the DAN family BMP antagonists include GREM1 (ENSJJAG00000013743) and DAND5 (ENSJJAG00000011222) which were not detectable in the Jerboa kidney, CER1 (ENSJJAG00000023511) which was not differentially expressed across conditions, NBL1 (ENSJJAG00000019434) which followed the pattern of GREM2, and SOST (ENSJJAG00000015417) displayed the opposite expression pattern to GREM2.

In the Jerboa kidney, BMP/TGFb signaling is inhibited during dehydration by an increase in receptor antagonist expression, and this continues even after water is made available and expression of antagonists return to control levels.

### SLC (solute carrier transporters)

SLCs are prevalent in the kidney.^39^ The need to reabsorb as much water as possible in the kidney during dehydration is intrinsically linked to the reabsorption of solutes. There were 264 genes named “SLC” detected in the Jerboa kidney, 44% (n = 133) of which were differentially regulated (padj < 0.01) in the RNAseq data (38 in dehydration, 120 in rehydration, and 25 in both). 248 SLC genes were included in the WGCNA analysis, and of these 59.7% were in the “turquoise” module.

**Figure 7:**
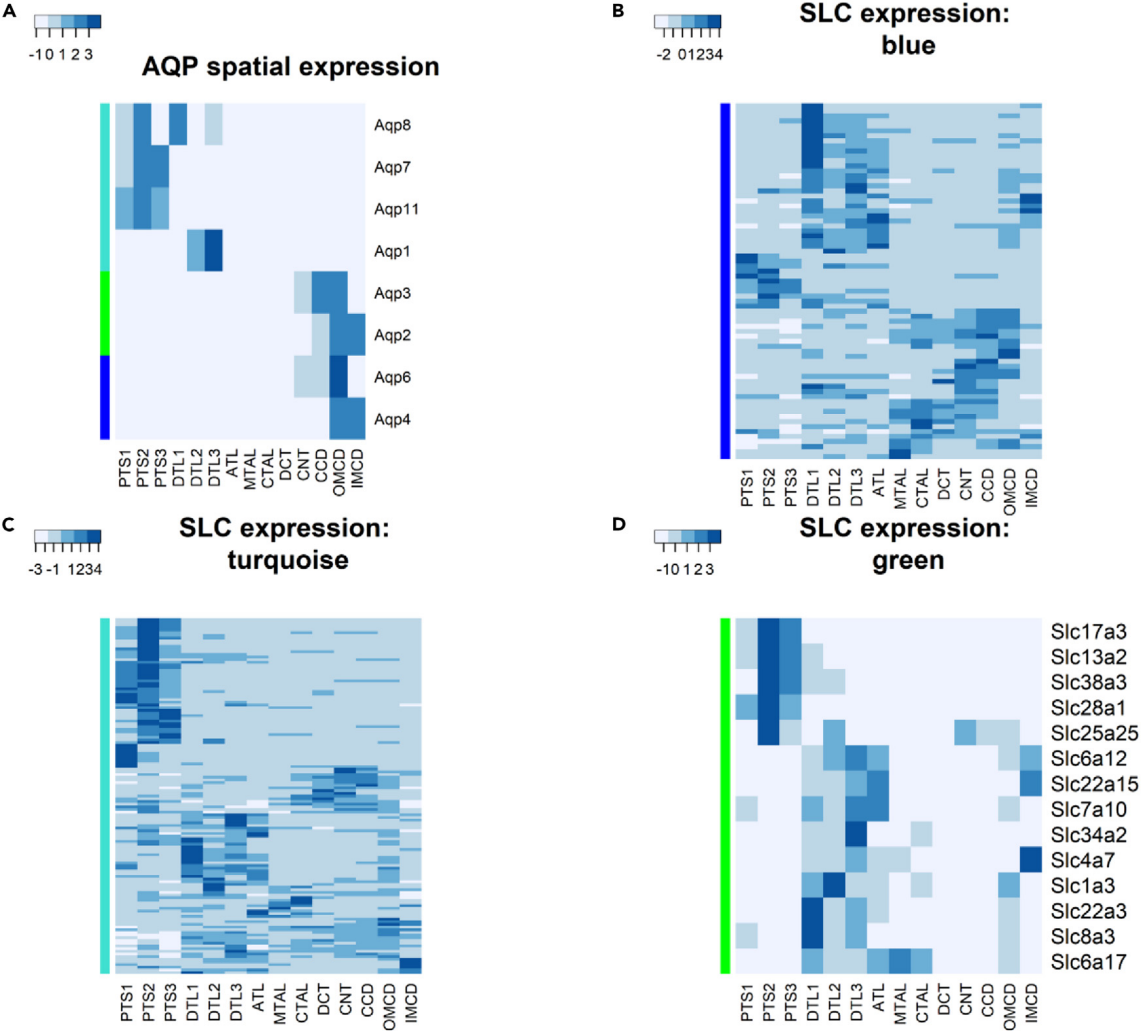
Spatial expression of genes expressing channels in the kidney Expression patterns of orthologue genes derived from the mouse by Chen et al.^40^ are combined with SLC genes specific to WGCNA modules of interest derived from the Jerboa expression data. This allows an approximation of expression of these solute channels along the nephron. (A) AQP expression is used as an example of how channels have spatial specificity in expression. (B–D) Expression of SLC genes in each module. Abbreviations taken from Chen et al.^40^ PTS1, the initial segment of the proximal tubule; PTS2, proximal straight tubule in cortical medullary rays; PTS3, last segment of the proximal straight tubule in the outer stripe of outer medulla; DTL1, the short descending limb of the loop of Henle; DTL2, long descending limb of the loop of Henle in the outer medulla; DTL3, long descending limb of the loop of Henle in the inner medulla; ATL, thin ascending limb of the loop of Henle; MTAL, medullary thick ascending limb of the loop of Henle; CTAL, cortical thick ascending limb of the loop of Henle; MD, mecula densa; DCT, distal convoluted tubule; CNT, connecting tubule; CCD, cortical collecting duct; OMCD, outer medullary collecting duct; IMCD, inner medullary collecting duct.

SLC genes are expressed throughout the kidney, reflecting their diverse targets. We deconvoluted our bulk RNAseq dataset to investigate where SLC genes were expressed in the Jerboa kidney and if this aligned with the expression patterns highlighted by the WGCNA modules. A transcriptomic map along renal tubules has been published using mouse tissue.^40^ We used this spatial data to match SLC genes in the Jerboa data to specific nephron sections (Figure 7). This excludes many of the cell types included within the whole Jerboa kidney (for instance, the glomerulus and blood vessels) but strong gene signatures may still be a useful signpost for spatial expression. To test the viability of this approach, the AQP family of genes was used (Figure 7A) and a clear tissue specificity was found using the mouse dataset.

SLC genes in the “turquoise” and “blue” modules were spread across most of the nephron segments (Figures 7C and 7E), likely due to the size of the modules capturing many different processes. Proportionally, the “turquoise” module contained more SLC genes with expression in the proximal tubules with a smaller number of genes expressed specifically in the descending limbs. The proximal tubule is involved in the bulk of solute reabsorption including sodium, glucose, potassium, calcium, and sodium bicarbonate so it is interesting that a number of these genes belong to the “turquoise” module with largely downregulated expression in dehydration and rehydration compared to control. Genes from the “turquoise” module specific to the proximal tubule included organic ion/anion transporters SLC22 (Nigam^41^) and sodium dependent glucose transporters SLC5 (Wright and Turk^42^). The opposite was true for genes in the “blue” module. Genes from the “blue” module found with high expression in the DTL1 (note that this “short loop” segment is devoid of AQP1 expression) segment included sodium-calcium exchangers of the SLC24 (Schnetkamp et al.^43^) and SLC8^44^ families.

We know that the “green” module contained genes such as AQP2 and AQP3 with high module connectivity and strong correlation with dehydration markers. These genes were expressed specifically in the medullary collecting duct segments (Figure 7A). Of the SLC expression in the “green” module that was specific to the inner medullary collecting duct, there were genes that have previously been shown to be osmoprotective (SLC6A12/BGT1/ENSJJAG00000018463). These are all processes that are linked to dehydration showing that the “green” module is representative across species. SLC genes in the “green” module also had a presence in the PTS2 segment. SLC17A3 (NPT4/ENSJJAG00000009931) secretes urate into tubules. High serum urate is linked to declining renal function, so this is perhaps an example of a protective mechanism in the Jerboa kidney.

### Limitations of this study

A potential issue related to correlations is the time course of expression. With a single time point snapshot for each experimental condition, some interactions may be lost as there will be a delay between expression of a gene (say a transcription factor) and the expression of downstream effectors.

The water restriction model used in this study is well established. The inclusion of the rehydration group is useful to show recovery of genes and therefore suggesting they are specific to osmoregulation rather than other potential mechanisms activated by dehydration. However, many genes had altered expression in both dehydration and rehydration compared to control. It is possible these genes may be activated/inhibited by acute stressors that are unrelated to the experimental conditions. These pathways and individual genes should be further investigated to define their functional relevance to osmoregulation. Furthermore, genes with reactive expression changes can be compared to those shown to be expressed differently between mesic and xeric species^45^^,^^46^ to elucidate sources of desert adaptation.

While a recent study estimated the completeness of the Jerboa genome at 92.8% by BUSCOs,^47^ the quality of the Jerboa genome annotation must be considered. The Jerboa genome is currently a scaffold assembly and gaps may reduce annotation efficacy. Over 1000 genes with Ensembl IDs had neither a gene symbol attached to them or a human orthologue that could be used with confidence. Many of these genes appear to belong to the mitochondrial genome.

An interesting possibility to consider is the effect of the acclimatization period. As the animals were wild there is no way of knowing what water availability was like before the acclimatization period. Therefore, it is possible that some animals in the control group could in fact be a pseudo rehydration sub-group.

Although RNA integrity number (RIN) values were all above 8.4, the rehydration group samples had lower values than the other two condition groups. However, a PCA plot of the RNAseq data (Figure S1A) shows a clear clustering of samples into the three experimental groups, suggesting that RIN is not one of the main drivers of expression differences between sample groups.

### Conclusions and perspectives

This study was designed to characterize the changes in blood analytes and renal transcriptome following dehydration in the desert rodent *Jaculus jaculus*. The experimental conditions triggered canonical responses in body weight, plasma sodium, and plasma osmolality.

The Jerboa kidney showed a large transcriptomic response to both dehydration and rehydration. Genes with high correlation to established dehydration responsive genes such as the water channel AQP2 warrant further functional investigation. For example, the increase in expression of GREM2, which is a BMP antagonist, coincides with an inhibition of BMP and TGF-beta receptor expression which continues even after rehydration. These mechanisms may be involved in the longer-term negative complications of dehydration in the kidney.

Rehydration is a hitherto understudied aspect of osmoregulation in desert rodents. The expression of many genes involved in translational machinery activated by water availability following a period of water restriction points toward a race to retain water opportunistically and offers interesting possibilities for the roles of ribosomal proteins to target specific mRNA transcripts in the kidney.

This study is the first step in understanding how the desert adapted Jerboas survive without water. This publicly available dataset is a resource that can be mined in future for questions regarding the transcriptional response to dehydration and rehydration in the desert adapted kidney and allows comparisons to be made with other datasets, both xeric and mesic. To this end we have made our data for multiple species accessible at app-shiny.services.bris.ac.uk/JerboaKidneyGeneSearch/.

## STAR★Methods

### Key resources table

**Table:** 

REAGENT or RESOURCE	SOURCE	IDENTIFIER
**Deposited data**		

Raw fastq files	This paper	GEO: GSE225470
Jaculus jaculus reference genome	Ensembl 99	GCA_00028075.1

**Oligonucleotides**		

Oligonucleotides for qPCR assay primers	See Table S2	N/A

**Software and algorithms**		

BBDuk	BBMap^48^	https://sourceforge.net/projects/bbmap/
FastQC	Andrews^49^	https://www.bioinformatics.babraham.ac.uk/projects/fastqc/
Rsubread	Liao^50^	https://bioconductor.org/packages/release/bioc/html/Rsubread.html
FeatureCounts	Liao^51^	https://subread.sourceforge.net/featureCounts.html
DESeq2	Love^52^	https://bioconductor.org/packages/release/bioc/html/DESeq2.html
gProfiler2	Raudvere^24^	https://biit.cs.ut.ee/gprofiler/page/r
WGCNA	Langfelder and Horvath^53^	https://cran.r-project.org/web/packages/WGCNA/index.html

### Resource availability

#### Lead contact

Further information and requests for resources and reagents should be directed to and will be fulfilled by the Lead Contact, David Murphy (d.murphy@bristol.ac.uk).

#### Materials availability

This study did not generate new unique reagents.

### Experimental model and study participant details

#### Animals

Wild *Jaculus jaculus* were caught in the area surrounding Riyadh, Saudi Arabia. All groups were collected from January-March 2019. Age of the animals cannot be reported as they were wild but they were considered adult. The average temperature in Riyadh at this time was 18.62°C with 0mm precipitation.^54^ Animals were singly housed at 25°C constant temperature, 12-hour light cycle (lights on at 0600), with free access to dry food pellets (EAN 5410340215906, Versele Laga) and water for an acclimatisation period of 7-13 days. The air handling system was designed to maintain relative humidity at a maximum of 15%. At the end of the acclimatisation period the average weight was 60.66g (43.3-76.5) for males and 64.28g (48.7-77.3) for females. From the first day in captivity, male animals increased their body weight by 11.6% and females by 7.7%. Animals were split into groups by sex and then randomly assigned to three experimental groups: euhydrated (control), dehydrated, and rehydrated. All animals were fed dry food. The euhydrated control group had access to water *ad libitum* for the duration of the study. The dehydrated group had their water source removed for 10 days for males and 10-11 days for females. The rehydrated group had their water removed for 7 days and then had access to water *ad libitum* for 4-5 days. Ethical approval for the collection of these samples was obtained from the University of Bristol (UB/18/085) and KSU Institutional Review Boards.

### Method details

#### Sample collection

To avoid stress to the wild animals caused by handling, animals were killed by ether overdose whilst still in their housing. Blood samples were collected by cardiac puncture and transferred to lithium heparin tubes. Kidneys were removed and rapidly frozen with dry ice. All samples were collected between 0830 and 1230 and stored at -80°C before transportation to the UK on dry ice under the authority of DEFRA import licence ITIMP18.0082.

#### Markers of dehydration

Blood analytes albumin (ALB), alkaline phosphatase (ALP), alanine aminotransferase (ALT), amylase (AMY), total bilirubin (TBIL), urea nitrogen (BUN), total calcium (CA), phosphorus (PHOS), creatinine (CRE), glucose (GLU), sodium (Na+), potassium (K+), total protein (TP), globulin (GLOB)) with haemolysis (HEM), lipemia (LIP), and icterus (ICT) quality control markers were measured in 100 μl of whole blood using a VetScanV2 (Abaxis, Union City, CA) with the Comprehensive Analysis Rotor. Whole blood was also analysed using a VetScan HM5 (Abaxis, Union City, CA) for haematology. If significant alterations of analyte concentrations were caused by haemolysis, icterus, and/or lipemia these results were supressed during processing by the VetScanV2 or HM5. In the absence of a direct measure of osmolality, calculated osmolality was estimated using the Worthley equation (2∗sodium (mmol/l)) + (glucose (mg/dL)/18) + (urea nitrogen (mg/dL)/2.8).^55^

### Quantification and statistical analysis

For each blood analyte, a General Linear Model (GLM) was built using a Gaussian family and Identity link as all dependant variables were continuous and the independent variables were a mix of continuous (eg haemolysis) and categorical data (eg sex). Using a GLM helps to control for effects of variables other than the experimental condition (control, dehydration, or rehydration groups) within the model. Samples were omitted in each case if data for the dependant variable was missing.

For each model the formula y ∼ Condition + Sex + Batch + Haemolysis + Lipemia was used where y is the blood analyte of interest. Sex specific differences have been identified in a variety of blood analytes^56^ and are thus is important to control for. Analyte results beyond acceptable limits of haemolysis and lipemia (>10% interference) were removed during processing by the VetScanV2 machine. The removal of results that did not meet the required standards altered the number of data points within each group and this is reflected in the reporting of the F statistic degrees of freedom. These variables can influence analyte results even when within acceptable limits, so they were also included in each model. Batch reflects the different dates that the animals were caught and therefore the different acclimatisation period lengths. Adherence to the assumptions (homoscedasticity, normal distribution, unduly influential observations) of each model was assessed visually.^57^ Models were reduced by stepwise removal of independent variables that contributed little to the model. Models were checked again and were then compared to the full model using Akaike's information criterion. The pwr.f2.test function in the pwr package^58^ in R was used to estimate the power of each model with a significance level of 0.05. A summary of each model including F statistic, degrees of freedom, and significance of difference from a null model is shown in Table S1. All dehydration markers analysis was performed in R 3.6.1^59^ and a significance threshold of P<0.05 was used throughout.

#### Tissue processing

The left kidney was ground to a fine powder using a pestle and mortar chilled with liquid nitrogen and stored in 1.5 ml tubes at -80°C. For RNA extraction, tissue was lysed by adding acid-guanidinium-phenol based reagent (Qiazol 79306, Qiagen) to each powdered tissue sample at a ratio of 1 ml per 100 mg of tissue, vortexed for 2 minutes, then incubated on ice for 10 minutes. Lysed samples were centrifuged at 12000xg for 10 minutes at 4°C and the supernatant was transferred to a new tube. 200 μl of chloroform (22711.244, VWR) was added to each sample and mixed by vortexing for 15 seconds. Samples were centrifuged at 12000xg for 15 minutes at 4°C. The RNA-containing upper aqueous phase was removed and mixed by pipetting with an equal volume of absolute ethanol. RNA was extracted using a Zymo Direct-Zol RNA miniprep kit (R2052) as per the manufacturer’s instructions. Total RNA concentration and 260/280 ratios were determined using a Nanodrop 2000c (Thermo Fisher Scientific). RIN values were calculated using BioAnalyzer 2100 (Agilent) with all sequenced samples returning RIN values >8.4.

#### Jerboa kidney RNAseq

RNA extracted from euhydrated, dehydrated, and rehydrated male Jerboa kidneys (n=5 per group) was sequenced following poly-A selection using Illumina HiSeq 4000 for ∼35 million reads per sample on a 75bp paired end run by Source BioScience. Reads were trimmed of adaptor sequences using BBDuk^48^ followed by FastQC^49^ quality control. Raw fastq files have been deposited under accession GSE225470. FastQC files were merged into one report using MultiQC.^60^ FastQC of trimmed fastq sequencing files showed consistent high-quality Phred scores. FastQC also reported <0.1% contamination of adaptor sequences and <1% of reads were made up of overrepresented sequences. Expected GC content confirmed the removal of sequencing adaptors by read trimming. A splice aware aligner, Rsubread^50^ was used to map reads to the publicly available *Jaculus jaculus* genome assembly GCA_00028075.1 downloaded from the Ensembl 99 database^61^ (ftp://ftp.ensembl.org/pub/release-99/fasta/jaculus_jaculus/dna/Jaculus_jaculus.JacJac1.0.dna.toplevel.fa.gz and it’s corresponding GTF file from ftp://ftp.ensembl.org/pub/release-99/gtf/jaculus_jaculus/Jaculus_jaculus.JacJac1.0.99.gtf.gz) using the default settings. Rsubread uniquely aligned an average of 84.1% (79.7%-85.7%) of paired end fragments to the genome, equivalent to 64 million read pairs (50.4m-71.9m). Mapped reads were summarised using FeatureCounts v1.6.2^51^ grouping to gene identifiers. Featurecounts assigned an average of 49.7% (45.5%-52.6%) of total reads to features in the Jerboa genome, equivalent to 19.2 million reads (14.1m-22.9m). The Jerboa genome has a complement of 24433 identified coding genes. RNAseq found 15048 genes were reliably detected from kidney samples after removing lowly expressed genes (mean count across all samples <8). DESeq2 v1.38.3^52^ in R v4.0.3^59^ was used to estimate differential expression of genes between conditions with an adjusted p-value of 0.01 used as a significance threshold. This threshold was chosen to minimise the chance of any false positives that may occur from variability using wild non-model animals. A PCA plot (Figure S1) using variance stabilised transformed expression data (Love et al.^52^) showed clustering into the condition groups. PC1, PC2, and PC3 contributed to 38%, 14%, and 10% of the data variance, respectively. The remaining PC groups contributed less than 5% each. Two samples plotted away from their respective clusters (CM01 and RHM01). Removing the two samples from the analysis increased the number of DEGs by ∼300 in the control vs dehydration comparison but decreased the number of DEGs by ∼650 in the control vs rehydration comparison. Further investigation showed increased expression of inflammatory genes in CM01 and RHM01 samples compared to the rest of the samples (Figure S1). For this exploratory study, we decided to include the two samples arguing that important genes involved in the dehydration and rehydration response would still be robustly changed due to the importance of this system to survival in the Jerboa. Some degree of variability in biological replicates is expected as wild animals were used with no control over confounding variables such as age or health. Venn diagrams and volcano plots were constructed using the VennDiagram v1.7.3 package^62^ and ggplot2 from the tidyverse v1.3.2 package^63^ respectively. Gene Ontology (GO) gene set enrichment analysis was performed using the gProfiler2 v0.2.1 package^24^ using a significance threshold of <0.01 padj and the Ensembl 104 database.

#### RNAseq validation by RT-qPCR

DEGs identified by RNAseq were validated by an independent method – namely reverse transcription (RT)-qPCR. All 42 animals were used in qPCR experiments, thereby adding power to the findings, and taking into account any differences between male and female animals. Total RNA (2μg) was converted to cDNA using GoScript Reverse Transcriptase (A5003, Promega) as per the manufacturer’s instructions. Primers were designed using Primer-BLAST^64^ targeting exonic regions of genes of interest. Reference genes CUL1 (ENSJJAG00000003559) and PPIA (ENSJJAG00000015423) were selected as the best combination pair (stability value = 0.048) using the NormFinder Excel Add-in.^65^ Primer sequences (with efficiencies %) are shown in Table S2. All qPCR assays used 300nM primer concentrations and PowerUp SYBR green master mix (A25742, Thermo Fisher Scientific). Relative abundance for each gene of interest was estimated using the delta delta Ct formula 2^–ΔΔCt^.^66^

#### Network analysis

To incorporate the expression of genes across all three experimental conditions, WGCNA v1.72-1^53^ was used. Expression values were transformed using the varianceStabilizingTransformation() function in the DESeq2 R package (Love et al.^52^). Due to the relatively small number of samples (n=15), the transcriptome expression data was filtered to mitigate the effect of noise and computational limitations. Starting with a full dataset of 15048 genes reliably detected in RNAseq, 30% of the least variable genes across all samples (calculated by median absolute deviation/median, cutoff value 0.12) were removed to leave a dataset of 10533 genes. There was minimal difference between PCA plots using pre and post filtered data, confirming that the filtered genes had expression that did not contribute to variability between samples and the main driver of expression was the experimental conditions.

#### WGCNA

WGCNA clusters genes into modules by Biweight mid-correlation (Bicor) of expression patterns and topological overlap matrix dissimilarity measures. The correlation data was signed and a β power of 8 was chosen as a soft threshold based on R^2^ and median connectivity. The resulting network from the working dataset of 10533 genes produced 5 modules (at a cut height of 0.989). Two similar modules (bicor >0.65) were merged into one for a final total of 4 modules ranging from 343 to 4969 genes (Figure S2). To determine which modules may be functionally relevant, bicor correlations were drawn between module eigengenes (an abstract gene representative of the module) and the blood trait data presented above (Figure 3A). Only analyte data from sequenced samples were used and any missing values were computed using the use = “pairwise.complete.obs” argument within the WGCNA::bicor() function in R. The weight of animals at capture and total blood protein act as negative controls for the module/trait analysis. Neither trait correlated with any modules and therefore were not influenced by the experimental design. ORA was then carried out using the genes in each of the modules against a background list of all genes detected in the RNAseq experiment (significance threshold <0.05 padj) and representative terms were highlighted (full enriched term lists can be found in the Data S1).

## Data Availability

• Bulk RNA-seq data have been deposited at Gene Expression Ominbus (https://www.ncbi.nlm.nih.gov/geo/) and are publicly available as of the date of publication. Accession numbers are listed in the key resources table. • This paper does not report original code. • Any additional information required to reanalyze the data reported in this paper is available from the lead contact upon request.

## Supplemental information

Document S1. Figures S1 and S2 and Tables S1 and S2

Data S1. Full list of enriched Gene Ontology terms found for genes within each module, related to Figure 3
