# Supplementary material for: Mobilisation of jerboa kidney gene networks during dehydration and opportunistic rehydration
Source: iScience. 2023 Aug 9;26(9):107574. doi: 10.1016/j.isci.2023.107574 (PMC10470305; doi:10.1016/j.isci.2023.107574)
Supplement: Document S1. Figures S1 and S2 and Tables S1 and S2 [file mmc1.pdf]

iScience, Volume *26*

Supplemental information

Mobilisation of jerboa kidney gene networks

during dehydration and opportunistic rehydration

Benjamin T. Gillard, Nabil Amor, Fernando Alvira Iraizoz, Audrys G. Pauža, Colin Campbell, Michael P. Greenwood, Abdulaziz N. Alagaili, and David Murphy

Supplementary Table: GLM Summary

	range	model	df	adj.rsq	Fstat	pvalue	power	sig.factors	Condition.pvalue	DH_change	DH_StdErr	RH_change	RH_StdErr
Caught_Weight	29,70	Caught_Weight ~ Condition + Sex	47	0.17	4.36	0.01	0.69	Sex	0.75	1.59	2.79	-0.59	2.87
Weight_Day7	43,78	Weight_Day7 ~ Condition + Sex + Haemolysis + Lipemia	37	0.26	3.91	0.01	0.78	Sex	0.17	4.84	2.61	3.80	2.84
Weight_Day14	43,77	Weight_Day14 ~ Condition + Sex + Haemolysis + Lipemia	38	0.20	3.21	0.02	0.64	Sex	0.76	1.07	2.69	-0.99	2.85
Weight_7DayDehydration	38,82	Weight_7DayDehydration ~ Condition + Sex + Haemolysis + Lipemia	38	0.50	9.60	0.00	1	Condition,Sex,Haemolysis	0.00	-10.89	2.69	-14.00	2.85
dissection.weight	40,84	dissection.weight ~ Condition + Sex	47	0.46	15.29	0.00	1	Condition,Sex	0.00	-17.19	2.58	-8.00	2.66
Albumin	3.4,6	Albumin ~ Condition	23	0.35	7.75	0.00	0.85	Condition	0.00	0.57	0.14	0.23	0.18
Alkaline_Phosphatase	17,290	Alkaline_Phosphatase ~ Condition + Sex + Haemolysis + Lipemia	37	0.18	2.87	0.03	0.55	Sex,Haemolysis,Lipemia	0.65	-22.20	23.96	-9.20	25.27
Alanine_aminotransferase	14,160	Alanine_aminotransferase ~ Condition + Sex + Haemolysis + Lipemia	27	0.09	1.63	0.19	0.19		0.07	5.98	12.36	-28.00	13.65
Amylase	74,931	Amylase ~ Condition + Sex + Haemolysis	39	0.27	5.04	0.00	0.86	Condition	0.01	-183.03	56.34	-59.00	56.58
Total_Bilirubin	0.1,2.1	Total_Bilirubin ~ Condition + Sex + Haemolysis + Lipemia	37	0.97	248.94	0.00	1	Haemolysis,Lipemia	0.58	0.01	0.03	-0.02	0.03
Urea_Nitrogen	4,128	Urea_Nitrogen ~ Condition	47	0.01	1.36	0.27	0.094		0.27	8.66	5.57	0.76	5.68
Total_Calcium	2.9,12	Total_Calcium ~ Condition + Sex + Haemolysis + Lipemia	33	0.19	2.75	0.04	0.51	Condition	0.03	0.04	0.53	-1.40	0.58
Phosphorus	1.3,12	Phosphorus ~ Condition + Sex	45	0.42	12.43	0.00	1	Condition,Sex	0.00	1.95	0.52	-0.72	0.52
Creatinine	0.1,0.6	Creatinine ~ Condition	41	-0.04	0.17	0.84	Adjusted R2 is negative. Power calculation impossible		0.84	0.03	0.05	0.03	0.05
Glucose	162,364	Glucose ~ Condition	11	0.03	1.18	0.34	0.068		0.34	6.65	36.12	49.00	34.06
Sodium	128,159	Sodium ~ Condition + Lipemia + Haemolysis	33	0.57	13.38	0.00	1	Condition,Haemolysis	0.00	4.15	1.41	-3.50	1.62
Potassium	5.4,12	Potassium ~ Condition + Sex + Haemolysis	28	0.59	12.57	0.00	1	Haemolysis	0.19	0.34	0.36	-0.43	0.39
Total_Protein	1.2,8	Total_Protein ~ Condition	47	0.10	3.76	0.03	0.46	Condition	0.03	-0.18	0.28	-0.78	0.29
Globulin	0.5,2.5	Globulin ~ Condition + Lipemia	16	0.62	11.55	0.00	0.99	Condition	0.00	-1.14	0.20	-0.48	0.28
Haemolysis	92,2080	Haemolysis ~ Condition + Sex + Haemolysis + Lipemia	39	0.15	2.85	0.04	0.48	Lipemia	0.54	-162.28	163.29	-160.00	173.46
Lipemia	117,3852	Lipemia ~ Condition + Sex + Haemolysis + Lipemia	39	0.33	6.35	0.00	0.95	Sex,Haemolysis	0.09	126.17	293.88	640.00	295.12
WBC	0.28,8	WBC ~ Condition + Sex + Haemolysis + Lipemia	28	0.07	1.46	0.23	0.15		0.11	-0.66	0.85	-1.30	0.62
LYM	0.33,4.5	LYM ~ Condition + Sex + Haemolysis + Lipemia	28	0.06	1.39	0.26	0.13		0.06	-0.48	0.52	-0.97	0.38
MON	0.01,0.61	MON ~ Condition + Sex + Haemolysis + Lipemia	28	-0.07	0.56	0.73	Adjusted R2 is negative. Power calculation impossible		0.33	-0.05	0.06	-0.07	0.04
NEU	0.04,4.6	NEU ~ Condition + Sex + Haemolysis + Lipemia	28	0.01	1.05	0.41	0.059		0.74	-0.12	0.55	-0.31	0.41
LY.	26,94	LY. ~ Condition + Sex + Haemolysis + Lipemia	28	0.05	1.36	0.27	0.12		0.44	-10.89	9.33	-6.80	6.86
MO.	0.5,9.4	MO. ~ Condition + Sex + Haemolysis + Lipemia	28	-0.09	0.48	0.79	Adjusted R2 is negative. Power calculation impossible		0.83	0.39	1.51	-0.46	1.11
NE.	1.5,66	NE. ~ Condition + Sex + Haemolysis + Lipemia	28	0.09	1.64	0.18	0.19		0.41	10.48	8.89	7.20	6.54
RBC	6.4,16	RBC ~ Condition + Sex + Lipemia	29	0.26	3.95	0.01	0.7	Condition	0.00	1.71	0.50	-0.16	0.37
HGB	7.7,25	HGB ~ Condition + Sex + Lipemia	29	0.45	7.78	0.00	0.98	Lipemia	0.05	1.46	0.72	-0.33	0.53
HCT.	11,54	HCT. ~ Condition + Sex + Lipemia	29	0.19	2.99	0.03	0.5	Condition	0.01	4.68	3.21	-5.10	2.37
MCV	28,39	MCV ~ Condition	38	-0.01	0.81	0.45	Adjusted R2 is negative. Power calculation impossible		0.45	-0.14	0.94	-0.88	0.70
MCH	12,19	MCH ~ Condition + Sex + Haemolysis + Lipemia	28	0.31	4.01	0.01	0.77	Sex,Lipemia	0.38	-0.85	0.66	0.01	0.49
MCHC	35,58	MCHC ~ Condition + Sex + Haemolysis + Lipemia	28	0.35	4.48	0.00	0.84	Sex	0.09	-1.11	1.79	2.30	1.32
RDWc.	19,-Inf	RDWc. ~ Condition	38	0.03	1.57	0.22	0.12		0.22	-1.22	0.93	-1.10	0.69
PLT	6,622	PLT ~ Condition + Sex + Haemolysis + Lipemia	28	0.01	1.09	0.39	0.065		0.16	-45.72	29.57	-41.00	23.56
PCT.	0.01,0.4	PCT. ~ Condition + Sex + Haemolysis + Lipemia	28	0.07	1.47	0.23	0.15		0.10	-0.05	0.03	-0.05	0.03
MPV	6.1,12	MPV ~ Condition + Sex + Haemolysis + Lipemia	28	0.17	2.35	0.07	0.39		0.07	-0.21	0.52	-0.97	0.41
PDWc.	23,34	PDWc. ~ Condition + Sex + Haemolysis + Lipemia	28	0.10	1.77	0.15	0.23		0.27	-1.68	1.05	-0.88	0.84
Calculated_Osmolality	273,374	Calculated_Osmolality ~ Condition + Haemolysis	34	0.29	6.13	0.00	0.87	Condition	0.00	11.66	5.00	-7.50	5.34

[a] Signif. codes: 0 '***' 0.001 '**' 0.01 '*' 0.05 '.' 0.1

Supplementary Table 1. Summary of models for analytes measured including weight, haematology, and biochemistry. Related to Table 1 and QUANTIFICATION AND STATISTICAL ANALYSIS in STAR methods.

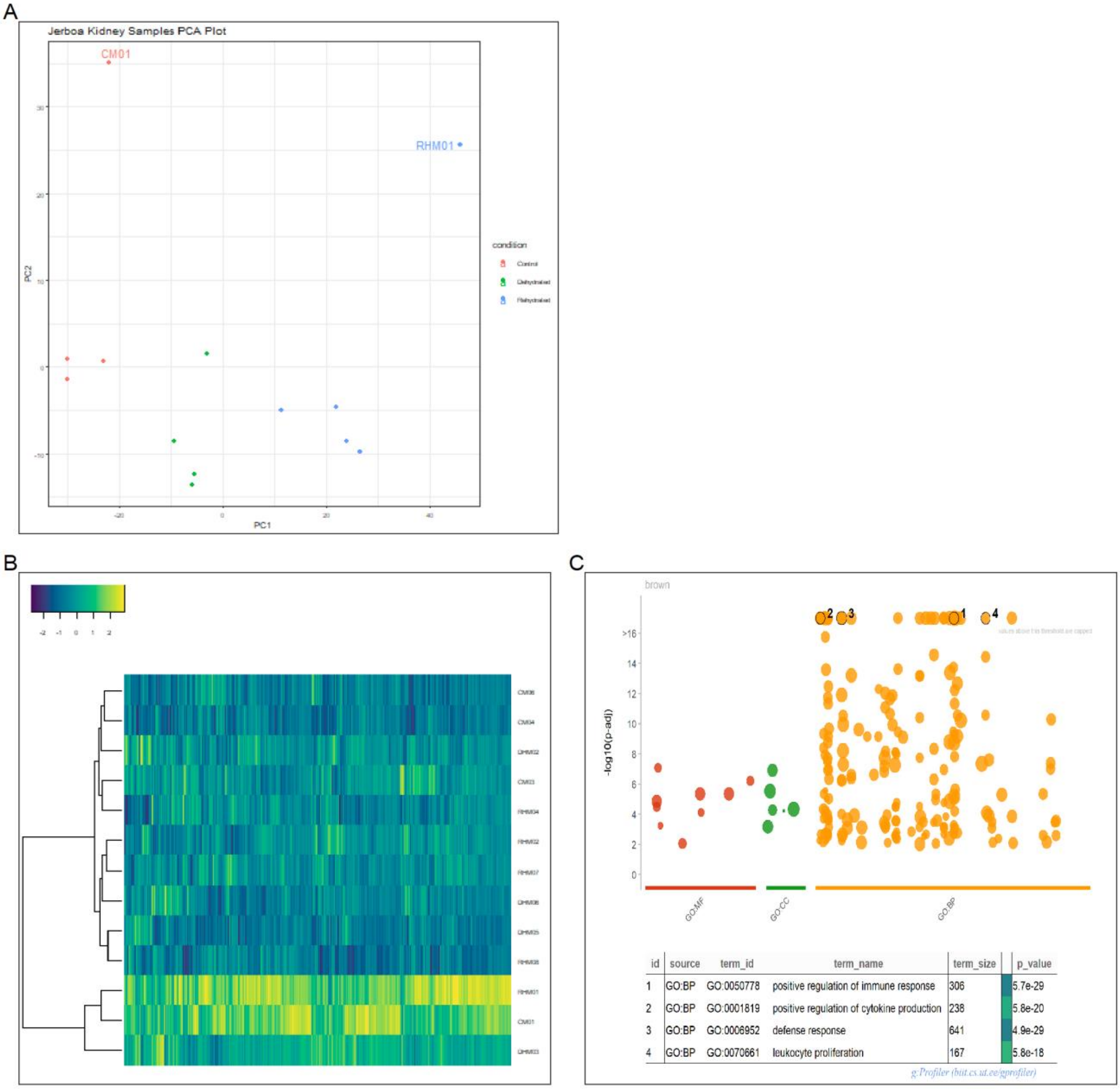

Supplementary Figure 1. Sample clustering and a description of the "brown" module when an outlier sample was included. Related to Jerboa kidney RNAseq section in STAR methods. A. PCA plot of sequenced Jerboa samples. Note the two samples that are not part of the clusters are CM01 and RHM01. B. A heatmap of gene expression within the "brown" module showed high expression in samples CM01, RHM01, and DHM03. C. The "brown" module was enriched for GO terms involved in the immune response.

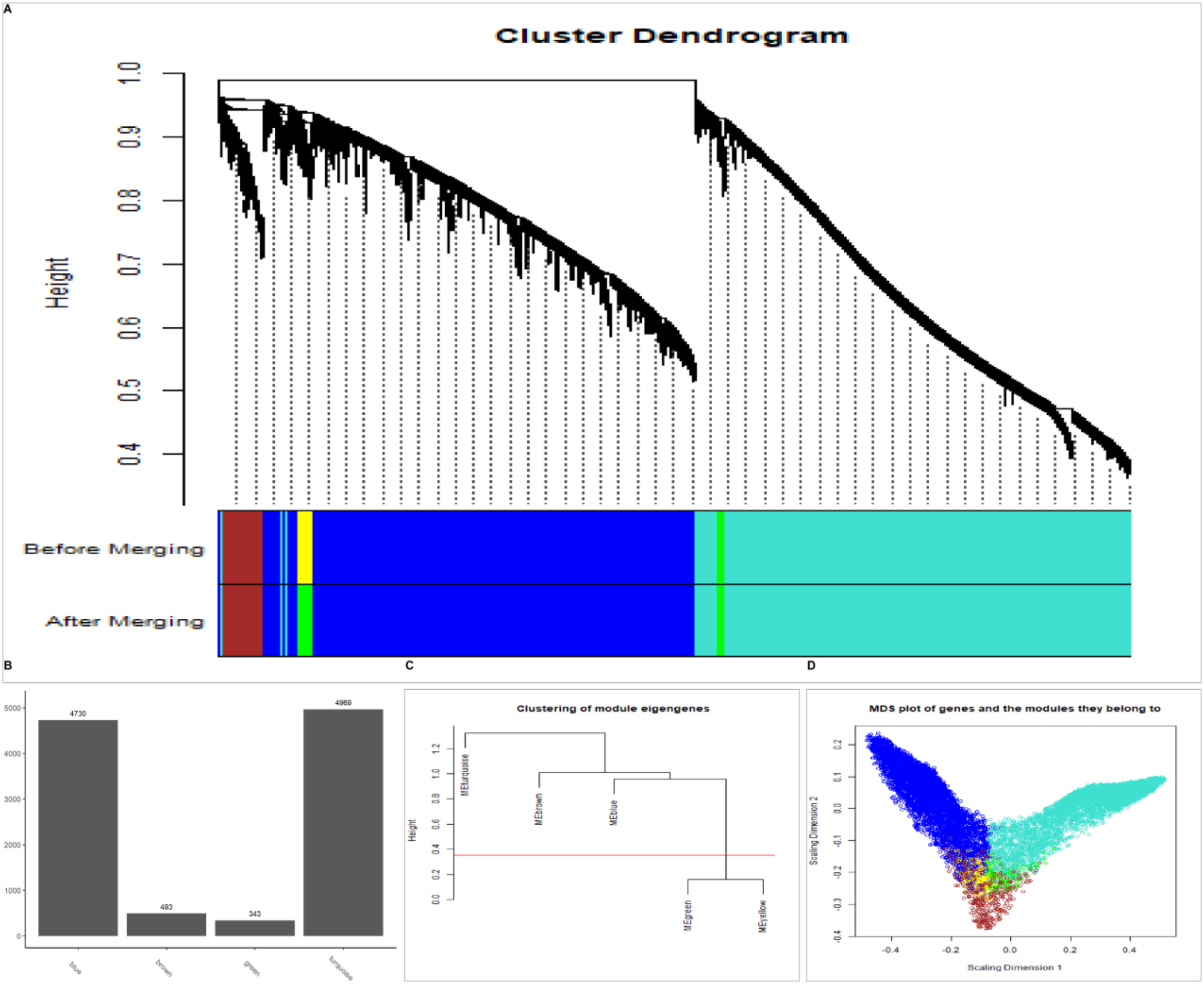

Supplementary Figure 2. Description of module building process using WGCNA, related to Figure 3 and WGCNA section in STAR methods. A. WGCNA created 6 modules from the filtered expression data. Clustering of module eigengenes shows how similar each module was to others. Modules “green” and “yellow” were deemed similar and were merged. B. Multi-dimensional scaling plot of all genes in the dataset and their assigned modules.

Primer Efficiencies

Gene	Ensembl_gene_id	F_Sequence	R_Sequence	Efficiency
AQP3	ENSJJAG00000004334	GGAGAGATGCTCCACATCCG	TGATGGTGAGGAAACCACCG	95.59
AQP2	ENSJJAG00000021696	ACTGGGTCTTCTGGATCGGA	GACGGGAACAGCACGTAGTT	100.84
AQP11	ENSJJAG00000023969	GGCTGGCTCCTTCTTTAGGG	TGCTTGGTTGTTATGCAGCC	105.37
AQP4	ENSJJAG00000010534	TCTGAAACCTGGAGTGGTGC	CCCGTCTGCTTTCAGTGCTT	106.06
AQP1	ENSJJAG00000017823	CTGGCGATCGACTACACTGG	CCAGGGCACCTCCAATGAAT	99.91
AQP6	ENSJJAG00000021101	GCTCTGCTTTATGGGGTCACT	GGTGCTGTTTTTGACCACGTT	93.15
AQP7	ENSJJAG00000011246	ACGGCCATTGAACAGTTTGC	TCCCGGTCAAAAACACCTCA	90.57
AQP8	ENSJJAG00000017882	TTGGGAAATATCAGCGGTGGA	CTGGGAGAGCCAGTAGGGAA	105.65
GREM2	ENSJJAG00000010680	CGAGCGCAAGTACCTCAAGA	CGCGGGATGTAGAAGGAGTT	96.10
SMAD5	ENSJJAG00000019065	CCGCCTATATGCCACCTGAG	GCAACAGGCTGAACATCTCG	99.89
SMAD7	ENSJJAG00000017342	ACCCTCATCACCTTAGCCGA	ATCTGGACAGTCTGCAGTTGG	95.17
TGFBR1	ENSJJAG00000008037	GGCCATTTGTATGTGCACCC	TCTGAAAAAGGGCCAGTCGT	96.74
TGFBR2	ENSJJAG00000009257	GGTCTATGACGATCCGCCAG	GCCTCCGTTTCCACATCAGA	103.34
TGFBR3	ENSJJAG00000017313	CCAGCCACAGAGAGAGGTTAC	GTCTTCAGACGCCAGACCAA	96.54
BMPR1A	ENSJJAG00000021938	TTCTCATGTCCAAGGGCAGAA	GGCAGTGTCCTGAGCAGTAG	110.04
BMPR1B	ENSJJAG00000012811	CAGTCAACAATATTTGCAGCACAG	AGAGGTGACAACAGGCGTTC	109.33
BMPR2	ENSJJAG00000015151	CTTACTGCCCAGTGTGCTGA	ATGTGACAGGTTGCGCTCAT	104.99
SCNN1A	ENSJJAG00000021122	GTGGTACCGCTTCCACTACA	AATGCGAGTAGTTTGCGTGG	92.84
SCNN1B	ENSJJAG00000022984	CAGCCCCTTCCAGTATTCCA	GGTGTGTGGTTCCAGATGGT	90.34
SCNN1G	ENSJJAG00000012195	GCACCCCAACTGGATGTACT	CTGACTGGCAGCCTAGCTTT	95.58

Supplementary Table 2. Primers used for each gene including unique gene identifier, sequences for forward and reverse primers, and primer efficiency. Related to Figures 5 and 6.
